# Nanomaterials in tumor immunotherapy: new strategies and challenges

**DOI:** 10.1186/s12943-023-01797-9

**Published:** 2023-06-13

**Authors:** Xudong Zhu, Shenglong Li

**Affiliations:** 1 Department of General Surgery, Cancer Hospital of Dalian University of Technology, Cancer Hospital of China Medical University, Liaoning Cancer Hospital & Institute, Shenyang, Liaoning 110042 People’s Republic of China; 2 Second Ward of Bone and Soft Tissue Tumor Surgery, Cancer Hospital of Dalian University of Technology, Cancer Hospital of China Medical University, Liaoning Cancer Hospital & Institute, Shenyang, Liaoning 110042 People’s Republic of China

**Keywords:** Nanomaterials, Tumor immunotherapy, Poly lactic-co-glycolic acid, Hydrogel nanoparticles, Liposomes, Lipid nanoparticles, Nanoemulsions

## Abstract

Tumor immunotherapy exerts its anti-tumor effects by stimulating and enhancing immune responses of the body. It has become another important modality of anti-tumor therapy with significant clinical efficacy and advantages compared to chemotherapy, radiotherapy and targeted therapy. Although various kinds of tumor immunotherapeutic drugs have emerged, the challenges faced in the delivery of these drugs, such as poor tumor permeability and low tumor cell uptake rate, had prevented their widespread application. Recently, nanomaterials had emerged as a means for treatment of different diseases due to their targeting properties, biocompatibility and functionalities. Moreover, nanomaterials possess various characteristics that overcome the defects of traditional tumor immunotherapy, such as large drug loading capacity, precise tumor targeting and easy modification, thus leading to their wide application in tumor immunotherapy. There are two main classes of novel nanoparticles mentioned in this review: organic (polymeric nanomaterials, liposomes and lipid nanoparticles) and inorganic (non-metallic nanomaterials and metallic nanomaterials). Besides, the fabrication method for nanoparticles, Nanoemulsions, was also introduced. In summary, this review article mainly discussed the research progress of tumor immunotherapy based on nanomaterials in the past few years and offers a theoretical basis for exploring novel tumor immunotherapy strategies in the future.

## Introduction

**Fig. 1:**
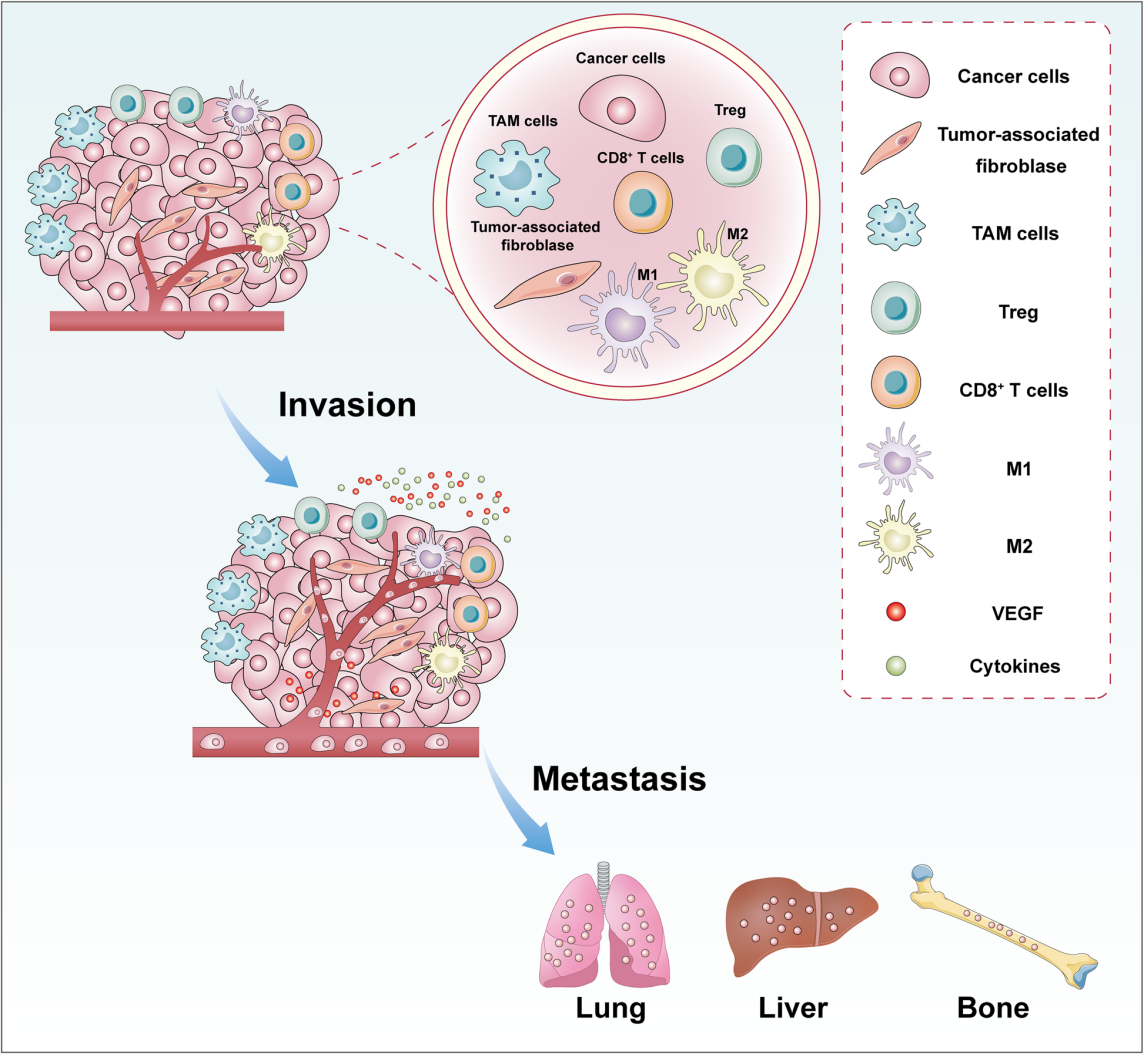
The basic conditions of tumor microenvironment. Tumor microenvironment consists of tumor cells, tumor-associated fibroblasts, tumor-associated macrophages (TAM), Treg cells, CD8 + T cells, M1 macrophages, M2 macrophages, VEGF, cytokines and other related immune cells, all of them collectively contribute to tumor invasion and metastasis

**Fig. 2:**
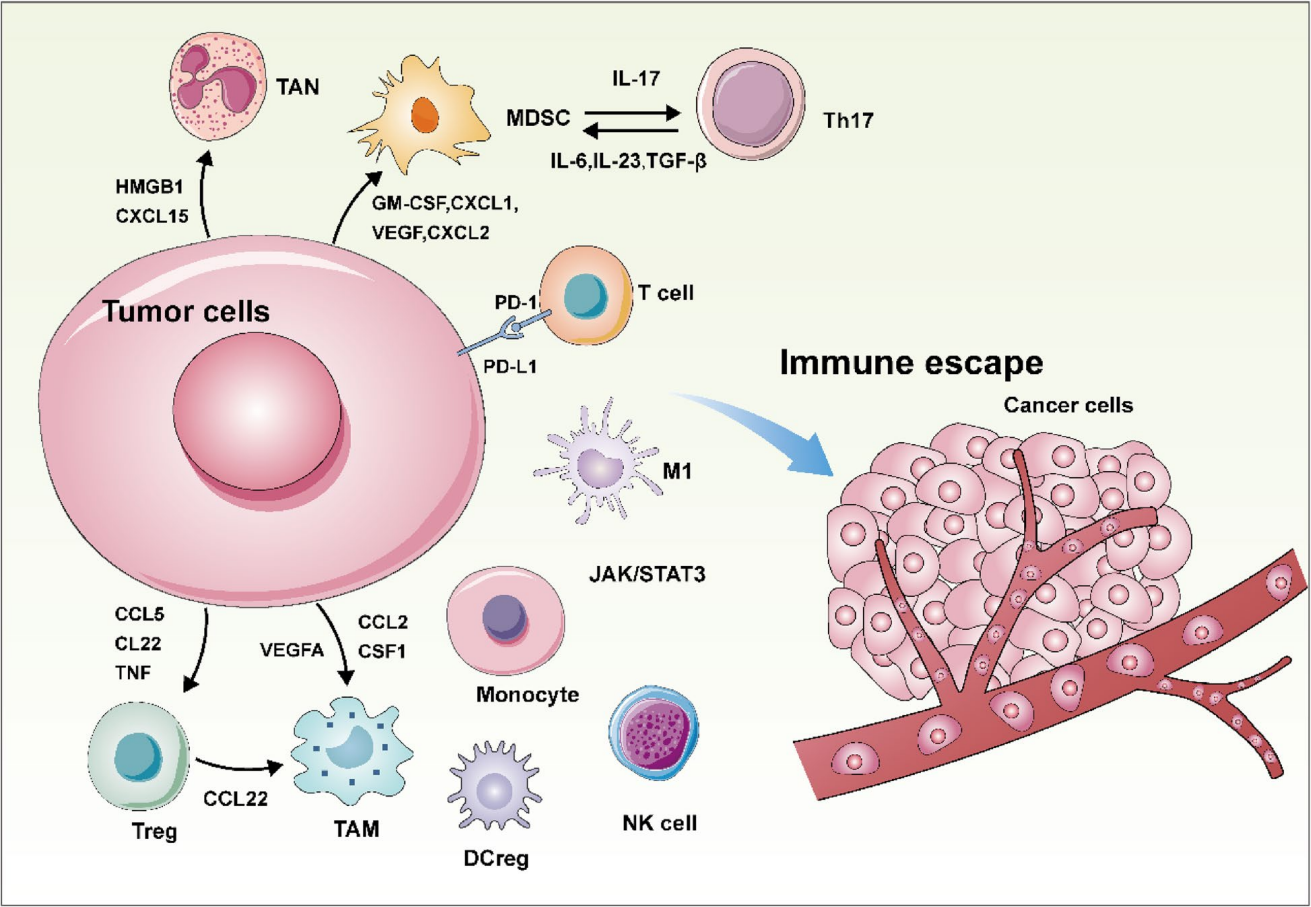
The actions between cancer cells and tumor microenvironment contribute to cancer immune escape. In tumor microenvironment, tumor cells secrete many kinds of chemokines and cytokines to inhibit the anti-tumor immune response by recruiting immunosuppressive cells. Specifically, tumor cells secrete CXCL15 and HMGB1 to recruit tumor-associated neutrophil (TAN) and secrete GM-CSF, CXCL1, VEGF and CXCL2 to recruit myeloid-derived suppressor cell (MDSC). MDSC act with T helper 17 cell (Th17) by IL-17, IL-6, IL-23 and TGF-β. Tumor cells secrete CCL5, CL22 and TNF to recruit regulatory T cell (Treg). Furthermore, tumor cells secrete VEGF, CSF1 and CCL2 to recruit tumor-associated macrophage (TAM). Treg cells further recruit TAM by secreting CCL22. In addition, tumor cells express and secrete responding proteins to interact with immune cells and inhibit their tumor-killing ability, as a result, promoting cancer immune escape. For example, overexpression of PD-L1 on tumor cells can interact with PD-1 expressed on T cells to deliver inhibitory signal. Meanwhile, tumor cells secrete extracellular vesicles, like exosomes which contains JAK/STAT3 pathway-related proteins, to surrounding tumor microenvironment. These proteins in tumor microenvironment interact with regulatory DC cell (DCreg), NK cells, Monocyte, and M1 macrophage to inhibit anti-tumor immune response and shape immunosuppressive microenvironment. As a result, immune escape happens in tumor cells

Malignant tumors are one of the most life-threatening diseases around the world [1, 2]. Although molecular typing and the emergence of comprehensive treatment plans have greatly improved the survival outcomes of patients [3], local recurrence and distant metastasis remain the leading factors that result in cancer-related death [4, 5]. Therefore, researchers are focusing their efforts on developing effective treatments that could inhibit tumor recurrence and metastasis. At present, surgery, chemotherapy, radiotherapy, targeted therapy and immunotherapy are the common modalities used in tumor treatment [6]. These treatment methods have yielded positive survival outcomes at the initial stage [7]. However, tumors often develop drug resistance with prolonged treatment, consequently leading to tumor recurrence and metastasis [8–10]. Tumor microenvironment (TME) plays an important role in tumor progression. TME consists of tumor cells, tumor-associated fibroblasts, endothelial cells, neurons, cytokines, growth factors, extracellular vesicles, Treg cells, CD8^+^ T cells and other related immune cells, all of which collectively contributes to tumor invasion and metastasis [11–17] (Fig. 1). Changes that take place within the TME is found to affect the effectiveness of immunotherapy besides contributing to cancer immune escape (Fig. 2), ultimately resulting in tumor progression and death [18–22]. The interactions between tumors cells and immune cells to promote invasion and metastasis of tumor by inhibiting anti-tumor immune response and inducing tumor immune escape within tumor microenvironment is summarized in Table 1.

**Table 1:** The interactions between tumors cells and immune cells to promote invasion and metastasis of tumor by inhibiting anti-tumor immune response and inducing tumor immune escape within tumor microenvironment

Cell types	Secreted chemokines, cytokines or proteins	Targeted cells	Results of interaction	Reference
Tumor cells	CXCL15, HMGB1	TAN	Inhibition of anti-tumor immune response and promotion of tumor immune escape	[23, 24]
Tumor cells	GM-CSF, CXCL1, VEGF, CXCL2	MDSC	Inhibition of anti-tumor immune response and promotion of tumor immune escape	[25–28]
Tumor cells	CCL5, CL22, TNF	Treg	Inhibition of anti-tumor immune response and promotion of tumor immune escape	[7, 29, 30]
Tumor cells	VEGF, CSF1, CCL2	TAM	Inhibition of anti-tumor immune response and promotion of tumor immune escape	[31–33]
Tumor cells	PD-L1	T cells	Inhibition of anti-tumor immune response and promotion of tumor immune escape	[34–36]
Tumor cells	JAK/STAT2	DCreg, NK cells, Monocyte, M1 macrophage	Inhibition of anti-tumor immune response and promotion of tumor immune escape	[37–40]
MDSC	IL-17	Th17	Inhibition of anti-tumor immune response	[41–43]
Th17	IL-6, IL-23, TGF-β	MDSC	Inhibition of anti-tumor immune response	[41–43]
Treg	CCL22	TAM	Inhibition of anti-tumor immune response	[7, 44]

**Fig. 3:**
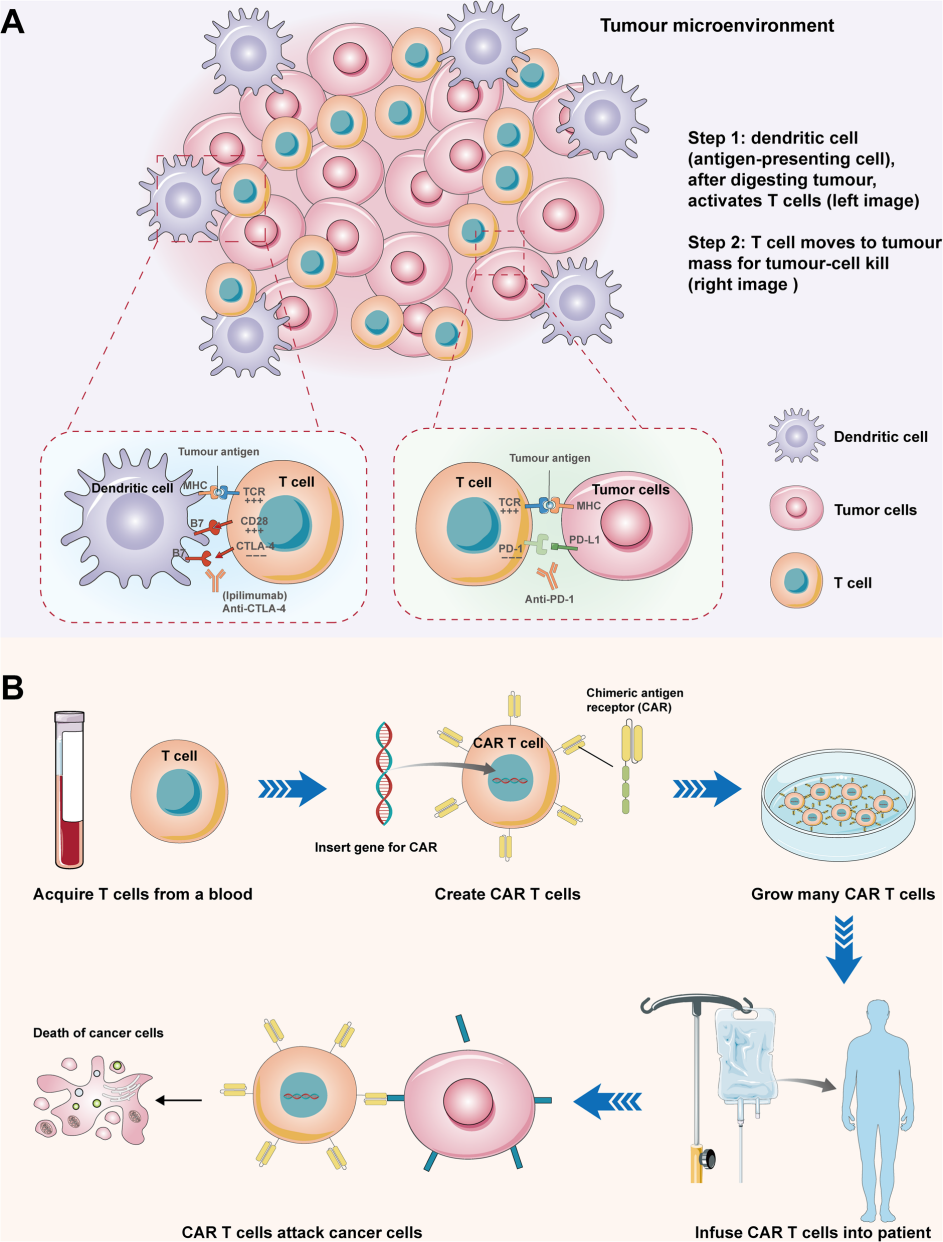
The utilization of immune checkpoint inhibitors such as the CTLA-4 antibody, the PD-1 antibodies, and the chimeric antigen receptor T cell (CAR-T) in tumor immunotherapy. **A**: The basic process of the CTLA-4 antibody and the PD-1 antibodies in tumor immunotherapy. Dendritic cells can activate T cells after digesting tumor cells in the tumor microenvironment. Then, T cells moves to tumor mass for tumor-cell killing. However, tumor cell can abrogate above process by CTLA-4 and PD-L1. For PD-L1, PD-L1 expressed on tumor cells can bind to PD-1 expressed on T cells. Then, this binding inhibited T cell activation. For CTLA-4, a naïve T cell-intrinsic inhibitor of T cell activation, can exert its function by trans-endocytosis of B7 (also called CD80/CD86) on dendritic cells. Then, naïve T cells lost the co-stimulatory signals from the interaction between CD28 on naïve T cells and B7 on dendritic cells. However, in this condition, naïve T cells receive signals by T cell receptor binding with its ligands, the complex of major histocompatibility complex antigen and peptide (MHC + P), which leads to T cell inactivation. As a result, tumor cells grow without the control of anti-tumor immunity. CTLA-4 antibody blocks the process of trans-endocytosis of B7 on dendritic cells and restore the tumor cell-killing ability of naïve T cells. Similarly, PD-1 antibodies restore the tumor-cell killing ability of T cells by preventing the binding between PD-L1 and PD-1. **B**: A schematic CAR-T therapy. The main processes of CAR-T therapy include obtaining T cells from blood, creating CAR-T cells, growing many CAR-T cells, and infusing CAR T cells into patient with cancer. At last, CAR-T cells can attack cancer cells

The types of cytokines produced in the TME could determine the result of tumor immunotherapy as they affect T cell infiltration and macrophage polarization [45–47]. The TME is usually hypoxic, thus affecting T lymphocyte infiltration, macrophage polarization, fibroblast proliferate and angiogenesis, making it hard to realize the ideal tumor-killing effect and for this, tumor cells may grow and metastasize [48, 49]. TME has been an important battlefield between the host immune system and the tumor as it always favor tumor-infiltrated lymphocytes [50]. Immunotherapy is a new kind of therapy that has rapidly progressed and experienced remarkable advances in the past decade [51–54]. Unlike conventional tumor treatment methods that target the tumor cells, immunotherapy works on the body's immune system instead. The tumor-immunity cycle includes the following steps: (1) generation of sufficient effector T cells in vivo; (2) these effector T cells then infiltrate into the tumor and overcome the inhibition of TME; (3) the direct recognition of tumor antigen by effector T cells generate anti-tumor immune response; and finally (4) the persistency of anti-tumor response and the increased number of effector T cells [55]. At the end of the cycle, tumor cells will be removed by the body’s enhanced immune responses, reshaping the TME [56–58]. In summary, tumor immunotherapy enhances immune response-mediated tumor cell lysis by inducing the production of relevant immune cells to effectively and directly recognize tumor cells via tumor antigens [59, 60]. Besides, tumor immunotherapy also reduces or inhibits immunosuppressive signals that are induced by TME or tumor cells [61]. Presently, the most widely used strategies in tumor immunotherapies that were proven to be effective involve the utilization of immune checkpoint inhibitors such as the CTLA-4 antibody, the PD-1 antibodies and the chimeric antigen receptor T cell (CAR-T), all of which had presented powerful anti-tumor activity in treating a variety of solid tumors and hematologic malignancies [15, 48, 62–64] (Fig. 3). A number of tumor immunotherapeutic drugs have successfully entered clinical application [16, 65–67]. However, in vivo tumor immunotherapy drugs still face several key challenges owing to poor tumor permeability and low tumor cell uptake. Firstly, the drugs may lead to the generation of specific T cells that cannot effectively and directly target tumor cells. Secondly, there is no success in eliciting continuous anti-tumor immune responses for long-term protective effect. Some studies further explored the potential mechanisms why immunotherapy drugs failed to generate T cells with specificity and efficiency and also proposed relevant methods to overcome such gaps.

Firstly, upon the use of immunotherapy drugs, the level of interferons and other inflammatory cytokines like IL-6, IL-12 or TGFβ significantly increased in the TME, which lead to the overexpression of PD-L1 on tumor-infiltrating immune cells. The cumulative interaction between PD-L1 and PD-1 contribute to the state of T cell dysfunction which often referred to T cell exhaustion and adaptive immune resistance. Therefore, these T cells lost the specificity and efficiency to kill tumor cells and the effect of immunotherapy drugs was poor [68–70]. For the proposed methods to overcome this gap, present studies have found that T cells also expressed the activating receptor CD226. CD226 can compete with the inhibitory receptors CD96 and T cell immunoreceptor with Ig and ITIM domain (TIGIT) expressed on T cells for binding to CD155 overexpressed in tumor cells to exert anti-tumor effect [71]. However, the exist of CD96 and TIGIT significantly inhibit the anti-tumor immune response of T cells [72–74]. The results from preclinical tumor models presented that therapeutic targeting of CD96 and TIGIT has demonstrated significant efficacy and synergistic activity with PD-1 inhibition, which could restore T cell exhaustion to a certain extent [75, 76]. Furthermore, under the immunotherapy drugs-used TME, T cells can also induce the production of macrophage colony-stimulating factor 1 (CSF1). CSF is a kind of key regulator of macrophage differentiation which maintains the pro-tumorigenic functions of tumor-associated macrophage to affect the specificity and efficiency of T cells on anti-tumor immune response indirectly [77]. Further clinical research results also found that patients who did not respond to single anti-PD-1 therapy could re-respond to treatment that combined CSF1R inhibitors and anti-PD-1 antibodies [77].

Additionally, in patients with chronic or advanced cancer, persistent antigen exposure induces sustained T cell receptor stimulation. Subsequently, T cell function continues to decline. Dysfunction T cells reduced the production of tumor-killing cytokines and overexpressed the inhibitory receptors like PD-1, CTLA-4 and LAG3 as well as immunosuppressive enzyme CD39. At last, T cell exhaustion happened [78, 79]. T cell exhaustion itself is also an important reason that immunotherapy drugs cannot generate and collect enough T cells with specificity and efficiency. Also, immunotherapy drugs cannot exert effective anti-tumor effects [80, 81]. The direct phenomenon is that single use of a kind of immunotherapy drug only showed weak response. To overcome this issue, the most effective method is the combinational use of medication. For example, compared with untreated patients with advanced melanoma, the combination of nivolumab (PD-1 antibody) and relatlimab (LAG3 blocking antibody) showed better clinical benefits than nivolumab alone, presented strong and synergistic effect on reversal of T cell exhaustion and has recently been approved for marketing [82–84]. Besides, combination of checkpoints blockers, combination of checkpoint blockade with costimulatory agonists, combination of checkpoint blockade with manipulation of soluble mediators, and combination of checkpoint blockade with CAR T cell therapy also have been proven to overcome such gaps [50, 85, 86].

**Fig. 4:**
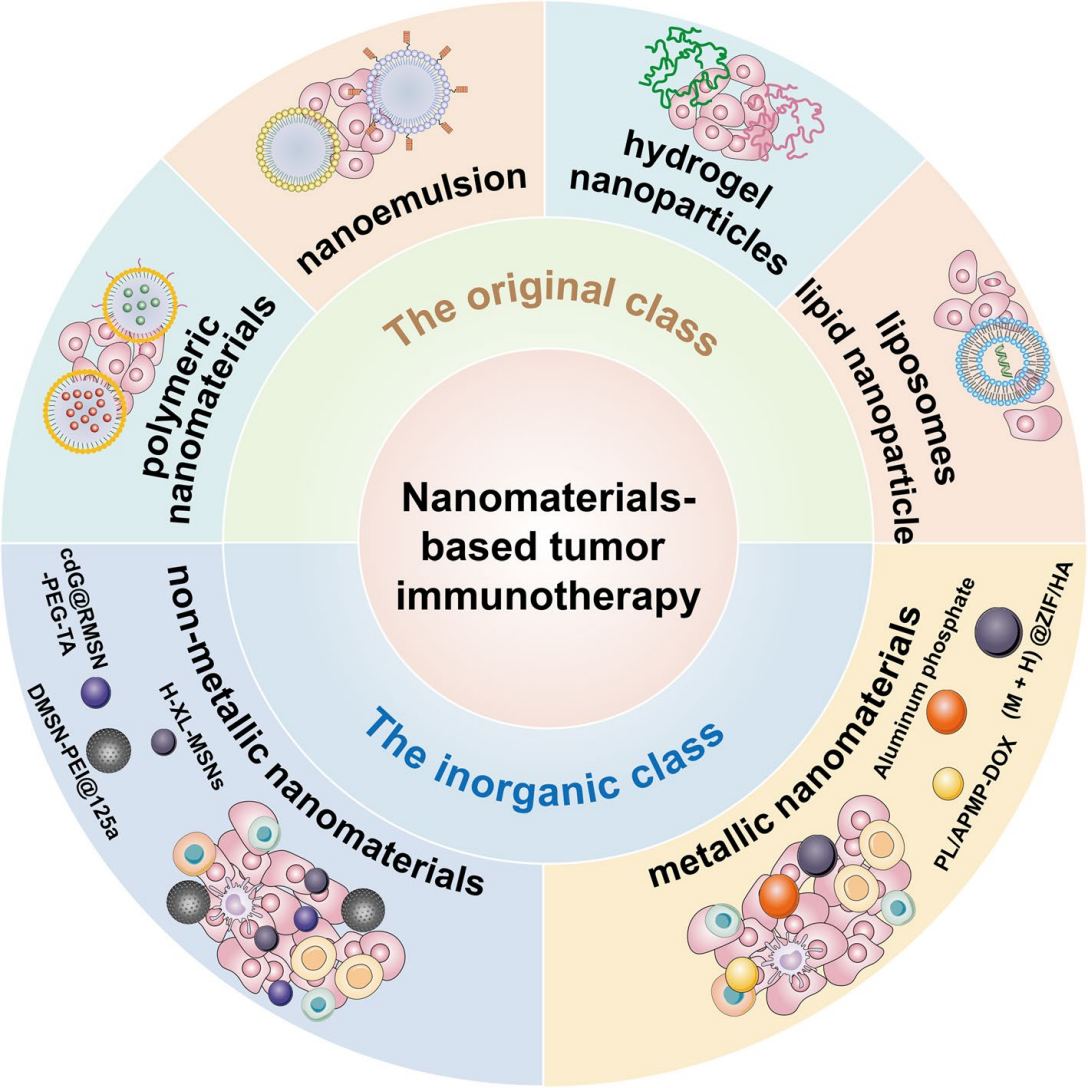
The different nanomaterial models and fabrication method for nanoparticles mentioned in this review. The common types of nanoparticles used in tumor immunotherapy that are discussed in this review, include organic class polymeric nanomaterials, nanoemulsion, hydrogel nanoparticles, liposomes and lipid nanoparticle; and inorganic class non-metallic nanomaterials and metallic nanomaterials

Nanomaterials refer to substances whose structural sizes are between 1 and 100 nm [87, 88]. Nanomaterials possess special thermal, biological and electromagnetic properties which are significantly different from general materials, such as surface effect, quantum size effect and macroscopic quantum tunneling effect [89–92]. An increased number of nanomaterials have been applied in cancer research [93–96], with many of them (such as metals and metal oxide nanoparticles) being found to exert unique anti-tumor properties, thus demonstrating the great potential of nanomaterials in tumor therapy [65, 97, 98]. Nanoparticles are divided into two main classes: organic (polymersome, dendrimer, polymer micelle, nanosphere, nano-hydrogel, liposome, and lipid nanoparticle) [99, 100] and inorganic (non-metallic nanomaterials, metallic nanomaterials and more) [101]. The common types of nanoparticles used in tumor immunotherapy that were mainly discussed in this review included polymeric nanomaterials (poly lactic-co-glycolic acid and hydrogel nanoparticles), liposomes and lipid nanoparticles from the organic class; and non-metallic nanomaterials and metallic nanomaterials from the inorganic class [102–107]. We also introduced the fabrication method for nanoparticles: Nanoemulsions. A schema of different nanomaterials model and fabrication method for nanoparticles mentioned in this review was shown in Fig. 4. This paper focuses on reviewing the research progress of tumor immunotherapy strategies that involve the aforementioned nanomaterials, with hopes that a breakthrough will be achieved in the near future.

## Tumor immunotherapy based on organic nanomaterials

### Polymeric nanomaterials

Natural and synthetic medicine-related polymer nanomaterials, for instance poly lactic-co-glycolic acid (PLGA), polyethylene glycol, polyurethane, polycaprolactone (PCL), poly(hydroxyacetic acid), hydrogel nanoparticles and others, are extensively applied in the field of tissue engineering, tissue regeneration, drug controlled release and tumor immunotherapy because of their excellent biodegradability, large surface area, low cytotoxicity and easy modification properties [108–112]. The different types of polymeric nanomaterials which have been reported were summarized in Table 2.

**Table 2:** The different types of polymeric nanomaterials

Types of polymeric nanomaterials	Reference
Poly lactic-co-glycolic acid	[113, 114]
Polyethylene glycol	[115]
Polyurethane	[116]
Polycaprolactone	[117]
Poly(hydroxyacetic acid)	[118]
Hydrogel nanoparticles	[119–121]
Polymersome	[122, 123]
Dendrimer	[124]
Polymer micelle	[125]

**Fig. 5:**
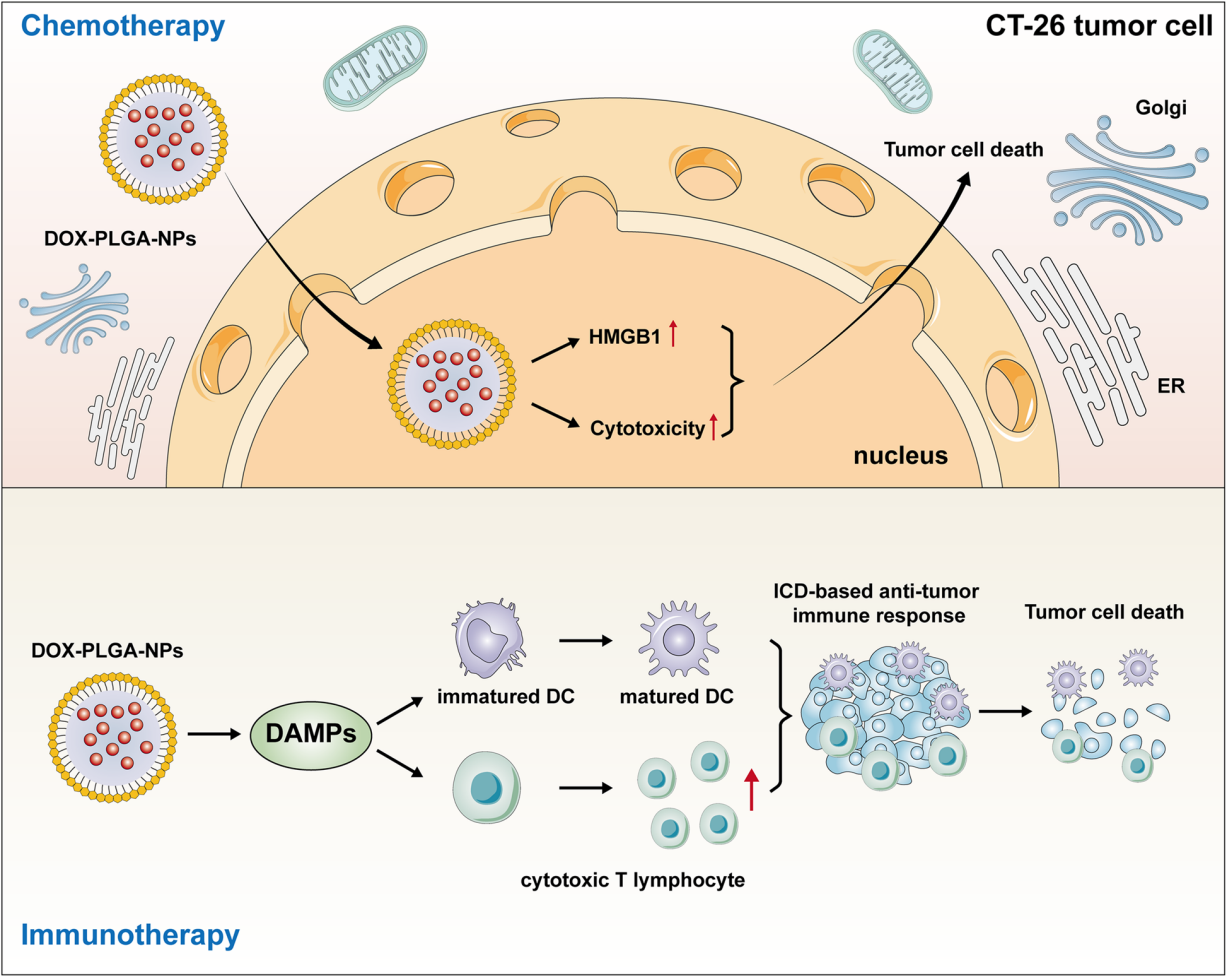
DOX-PLGA-NPs against tumor by inducing effects of chemotherapy and immunotherapy. DOX-PLGA-NPs, a kind of immunogenic cell death (ICD) inducer, can be introduced into CT-26 tumor cells and inhibit tumor growth in vivo by eliciting cytotoxicity and HMGB1 release. Besides, DOX-PLGA-NPs stimulate a tumor cell-specific immune response by releasing damage-associated molecular patterns (DAMPs), which promotes DC maturation and increases cytotoxic T lymphocytes tumor infiltration, causing an ICD-based specific anti-tumor immune response, finally, tumor cell death

Among all kinds of polymeric nanomaterials, PLGA is a kind of synthetic polymer-based nanocomposites with benign biocompatibility and high degradability. It has the advantages of slow controlled release, protecting DNA plasmid loop from breaking, high carrying capacity of encapsulation and the ability to escape from lysosome degradation as a kind of DNA vaccine carrier [113, 126–128]. Soodabeh et al. found that PLGA nanoparticles (PLGA-NPs) containing tumor lysate antigens could be obtained from fresh breast tumors. It was confirmed by relevant experiments that PLGA-NPs can be applied as tumor antigen delivery vectors to effectively stimulate the maturation of dendritic cells (DCs). PLGA-NPs can also perform targeted delivery and slow controlled release of tumor antigen [129]. By double solvent evaporation technique, DCs can be loaded into soluble tumor lysates which were encapsulated in nanoparticles to serve as a kind of DC vaccines. Furthermore, the NP-encapsulated antigens can bias the anti-tumor immune response of T cell towards Th1, then, it can enhance the effect of these DC vaccines [130]. Zhenzhen Chen et al. loaded ovalbumin and copper sulfide into PLGA-NPs to form nanocomplexes (cuS@OVA-PLGA-NPs) of core–shell structure. They found that cuS@OVA-PLGA-NPs could activate CD8^+^ T cells and induce anti-tumor immune response by induce IL-6, IL-12 and TNF-α expression. This result indicated that cuS@OVA-PLGA-NPs could be a kind of therapeutic nanomaterial for tumor immunotherapy [131, 132]. Yongwhan Choi et al. prepared PLGA-NPs with a variety of molecular weights. Then, they loaded doxorubicin (DOX) into these PLGA-NPs and form DOX-PLGA-NPs with different controlled release kinetics. Pre-clinical experiments showed that DOX-PLGA-NPs, a kind of immunogenic cell death (ICD) inducer, can be introduce into CT-26 tumor cells and significantly inhibit CT-26 tumor growth in vivo by cytotoxicity and HMGB1 release, and by inducing ICD. The reason that inducing ICD may be DOX-PLGA-NPs nanodrugs-related controlled release kinetics might significantly stimulate a tumor cell-specific immune response by releasing damage-associated molecular patterns (DAMPs), resulting in a different antitumor response in vivo. Additionally, they found that the immunological memory effect was also successfully established by the ICD-based specific anti-tumor immune responses in a mouse tumor model, including promoting DC maturation and the increase of cytotoxic T lymphocytes tumor infiltration. The controlled release of ICD-inducible, nanomaterials loaded chemotherapeutic agents may provide high potential in the development of precision cancer immunotherapy by controlling the tumor-specific immune responses, thus significantly improving the therapeutic efficacy in clinical practice [133]. These findings were illustrated in Fig. 5.

**Fig. 6:**
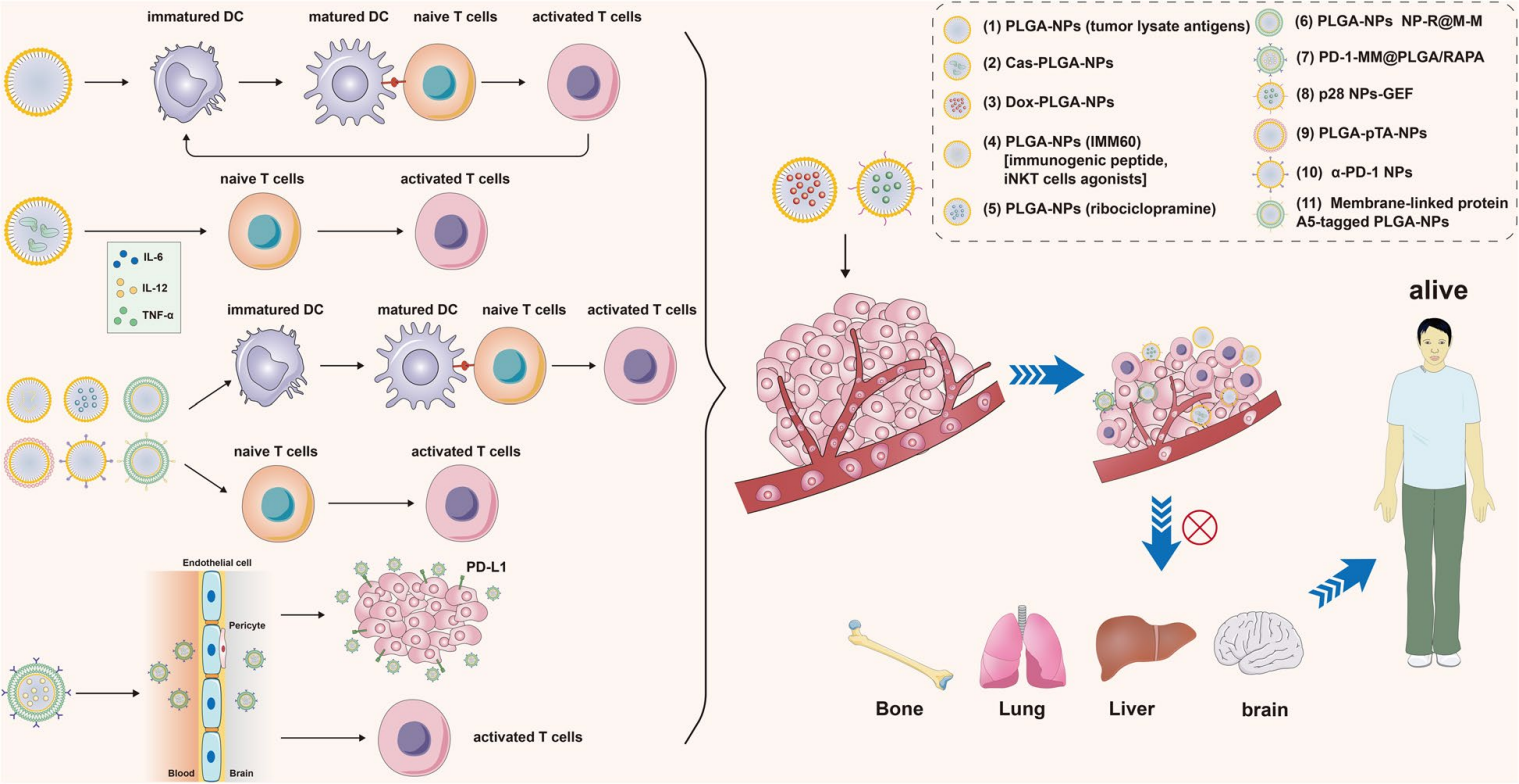
A summary of some kinds of PLGA NPs used in tumor immunotherapy to inhibit tumor progression. The 11 kinds of PLGA NPs mentioned in this manuscript activate T cells and restore T cell-induced tumor immunity to inhibit tumor progression

Yusuf Dölen et al. formed a PLGA-NP which is encapsulated with cancer-testis antigen NY-ESO-1 and α-GalCer analog IMM60. This PLGA-NP can enhance the anti-tumor immune response of CD4^+^ and CD8^+^ T cell and the antibody level against NY-ESO-1. Besides, This PLGA-NP can also stimulate the maturation of DCs and induce antigen-specific anti-tumor immune response of T cells by enclosing immunogenic tumor-related peptides and iNKT cell agonists to promote tumor immunotherapy [134]. Wenfeng Lin et al. applied PLGA to the fluorescent dye indocyanine green and the toll-like receptor 7/8 agonist (R848) to form a PLGA-ICG-R848 drug nanocarrier by the double solvent evaporation technique and found that PLGA-ICG-R848 nanoparticles presented anti-tumor effects when combining PLGA-pTA-based photothermal therapy (PTT) with anti-tumor immunotherapy [135]. Julia Koerner et al. encapsulated the ribociclopramine into PLGA-NPs vaccines. Then, they found these vaccines can significantly induced the maturation of DCs and the anti-tumor immune response of CD8^+^ T cells. Finally, these vaccines inhibited the growth and metastasis of tumor and improve the survival outcomes of patients with tumor [136, 137]. Zhang et al. combined PLGA with TLR-7 agonist R837 and wrapped this nanomaterial with the B16-OVA cell membrane to obtain NP-R@M [138, 139]. NP-R@M-M was further synthesized by modifying mannose that binds to antigen-presenting cells on the surface of NP-R@M. Results obtained demonstrated the ability of NP-R@M-M to activate innate immune escape by promoting DC uptake and maturation. At the same time, Zhou et al. wrapped R@P-IM nanoparticles which were also loaded with TLR7 agonist R837with cancer cell membrane [140]. Not only could R@P-IM NPs activate immune reaction in DCs and exert anti-tumor immunity function, the R@P-IM NPs can also reactivate the immune response of cytotoxic T lymphocyte and induce tumor ablation. As a result, R@P-IM NPs significantly enhanced the efficacy of tumor immunotherapy [136, 141, 142]. A novel nanoparticle called PD-1-MM@PLGA/RAPA was synthesized by Wang et al. by wrapping RAPA-loaded PLGA and PD-1 overexpressed macrophage membrane [143]. The results of in vivo experiments showed the ability of PD-1-MM@PLGA/RAPA to easily pass the blood–brain barrier, and its enrichment within close proximity to tumors where PD-L1 is generally highly expressed. Besides, PD-1-MM@PLGA/RAPA also induces the infiltration of CD8^+^ T cells, and enhances anti-tumor immune responses by releasing key regulators of inflammation such as TNF-α. As a result, PD-1-MM@PLGA/RAPA can effectively inhibit tumor growth. Ana Rita Garizo et al. functionalized PLGA NPs with p28 to deliver gefitinib (GEF). The delivery of p28 NPs-GEF can significantly reduce the metabolic activity of A549 lung cancer cells and then inhibit the growth and lung metastasis of A549 tumor cells [144]. A kind of mannan-modified PLGA NPs which contained polyribonucleoside polycytidylic acid and tumor cell lysates was used as a tumor antigen delivery tool to induce anti-tumor immune response in mice with breast tumor. This kind of PLGA NPs can significantly inhibit growth of breast cancer and present excellent anti-tumor effect in vivo [145]. For photothermal immunotherapy, a kind of poly (tannic acid) (pTA)-coated PLGA-NPs (PLGA-pTA-NPs) was synthesized. When used in 4T1 breast cancer cells, PLGA-pTA-NPs exhibit excellent photothermal conversion efficiency, benign photostability and strong photothermal cytotoxicity to inhibit tumor growth. When used in PTT, PLGA-pTA-NPs significantly trigger DC maturation by increasing the release level of DAMP, then tumor growth, invasion and metastasis can be inhibited [95, 144]. Christina K Lee et al. discovered that the delivery of anti-PD-L1 via PEG-PLGA polymers enhance clinical efficacy and reduce toxicity by increasing bioavailability and decreasing immune clearance [146]. They incubated α-PD-L1 F(ab)-PEG-PLGA polymer in an oil-in-water emulsion to form α-PD-L1 F(ab-PEG-PLGA nanoparticles (α-PD-L1 NPs), and found that not only do the engineered α-PD-L1 NPs prolong α-PD-L1 antibody circulation and reduce renal clearance, they also maintain anticancer activity and enhance immune activation [144]. Xu-Dong Tang et al. united PLGA-NPs with Toll-like receptor 3 and 7 ligands. Then, this PLGA-NPs were encapsulated heparinase CD4^+^ and CD8^+^ T cell epitopes or DEC-205-targeted combinatorial epitope peptides to induce anti-tumor immune response [147]. Parisa Badiee et al. constructed a PEG-PLGA nanoparticle contained GSK3 small molecule inhibitor SB415286. This PEG-PLGA nanoparticle remarkably decreased the expression of PD-1, and promote the proliferation and survival of CAR-T cells which were co-cultured with related tumor cells [148]. Shulan Han et al. prepared a novel PLGA-NPs which co-encapsulated with PLB, DIH and NH4HCO3. This novel PLGA-NPs could increase the targeting ability of tumor, restore the anti-tumor immune response and reverse the chemotherapeutic immunosuppressive effects under the condition of immunosuppressive TME. Besides, PLGA-NPs encapsulated with low doses of PLB and DIH remarkably ameliorate the survival outcomes of mice with hepatocellular carcinoma (HCC). Therefore, this agent can also become a therapeutic target for patients with HCC [149]. Sung Eun Lee et al. designed and developed a selective nanocarrier system which was consisted of membrane-linked protein A5-tagged PLGA-NPs. Then, the membrane-linked protein A5-tagged PLGA-NPs was encapsulated with tumor-specific antigens to stimulate anti-tumor immune response by increase the level of IFN-γ and CD8^+^ T cells [150]. Piyush Kumar and Rohit Srivastava prepared a novel PCL glycol chitosan (GC)-polysaccharide co-blended nanoparticles (PP-IR-NP) encapsulated with highly biocompatible and biodegradable monodisperse IR 820 for imaging purposes and effective PTT. The cytotoxicity to breast cancer cells was significantly enhanced upon treatment with PP-IR-NP, thus inhibiting cancer progression [151]. A summary of the above-mentioned PLGA NPs used in tumor immunotherapy to inhibit tumor progress was shown in Fig. 6.

Hydrogel nanoparticles are another kind of polymeric nanoparticles at the sizes of 1 ~ 1,000 nm with the following characteristics: benign biocompatibility, highly structural designability, high water retention and biodegradability. They are made up of internal cross-linked structures, and water is used as their main dispersion medium [152, 153]. Hydrogel nanoparticles have huge favorable properties: volume effect, surface effect, interfacial effect, permeation effect, size effect and more. These advantages favor them to obtain a lot of active sites for coupling with various functional components [119, 154, 155].

Jiana Jiang et al. constructed the CpG-MCA nanogel, a new immunogenic DNA hydrogel nanoparticle consisting of tandem repeats in their CpG units. This specific type of nanohydrogel can be used to against degradation, and contribute to the growth and survival of RAW264.7 cells by increasing TNF-α and IL6 expression. CpG-MCA nanohydrogels can be effective anti-tumor immunostimulants [156]. Besides, the tumor environment became acidic after operation, which could possibly promote macrophage polarization from M1 to M2 [157, 158], which would subsequently lead to tumor immune escape [159]. So, Gu et al. wrapped hydrogel spray A component contained αCD47@ CaCO3 and B component contained thrombin which is mixed CD47 antibody and fibrin gel [160]. Upon the combination of components, A and B, this nanoparticle can now induce M1 polarization of macrophages and promote phagocytosis by inhibiting the “Do not eat me” CD47-SIRPα pathway. On top of that, in vivo experiments showed downregulation of immunosuppressive cells (such as MDSCs and Treg cells) and transcription factors (such as HIFα) when the A and B components of hydrogel spray were combined [161, 162]. Sun et al. also constructed a dual-lumen nano-hydrogel spray to inhibit the recurrence and metastasis of melanoma by activating immune responses of the body [163]. Hydrogel spray tube A contains fibrin and α-PD-L1, whereas tube B contains thrombin and PexD. PexD is a platelet-derived extracellular vesicle (Pex) loaded with DOX. The combination of contents from both tubes of the hydrogel spray creates a gel in situ at the incision site. This gel functions as a drug release reservoir, releasing high concentrations of PexD and α-PD-L1 into the incision site to ensure entry into the blood. Not only does the released PexD promote antitumor immune responses, it also tracks and adheres to CTCs while α-PD-L1 blocks the PD1/PD-L1 pathway. As a result, this hydrogel spray inhibits tumor growth and prolongs survival of tumor-bearing mouse.

Yang et al. also constructed a hydrogel scaffold called the MRD hydrogel [164]. MRD hydrogel decreases the size of primary tumor while activating anti-tumor immunity. Results from in vitro experiments showed that MRD hydrogel induces necrosis in tumor cells and promotes the maturation of DCs. Meanwhile, results from in vivo experiments suggested the ability of this MRD hydrogel in prolonging the survival of B16-F10 tumor-bearing mouse and inhibiting tumor growth by promoting the maturation of DCs, activation of NK cells, inducing the polarization of M2 macrophage to M1 macrophage, and increased infiltration of cytotoxic T cells and effector memory T cells. Besides, MRD hydrogel also exhibits a high level of biosafety and biocompatibility. Rho-associated kinases (ROCKs) are key signaling molecules associated with tumor proliferation and metastasis. The ROCKs are also involved in the process of phagocytosis by macrophage [165, 166]. Chen et al. wrapped PPP (PLGAPEG-PLGA) [167] and Y27632 (ROCKs inhibitor) [168]. After it is injected into a tumor, a hydrogel scaffold state would form under radiofrequency ablation treatment at high temperature. Radiofrequency ablation treatment collects many antigen-presenting cells while the hydrogel scaffold releases Y27632, activating phagocytosis and enhancing immune responses of the body. High level of T cell infiltration also happens during this process.

**Fig. 7:**
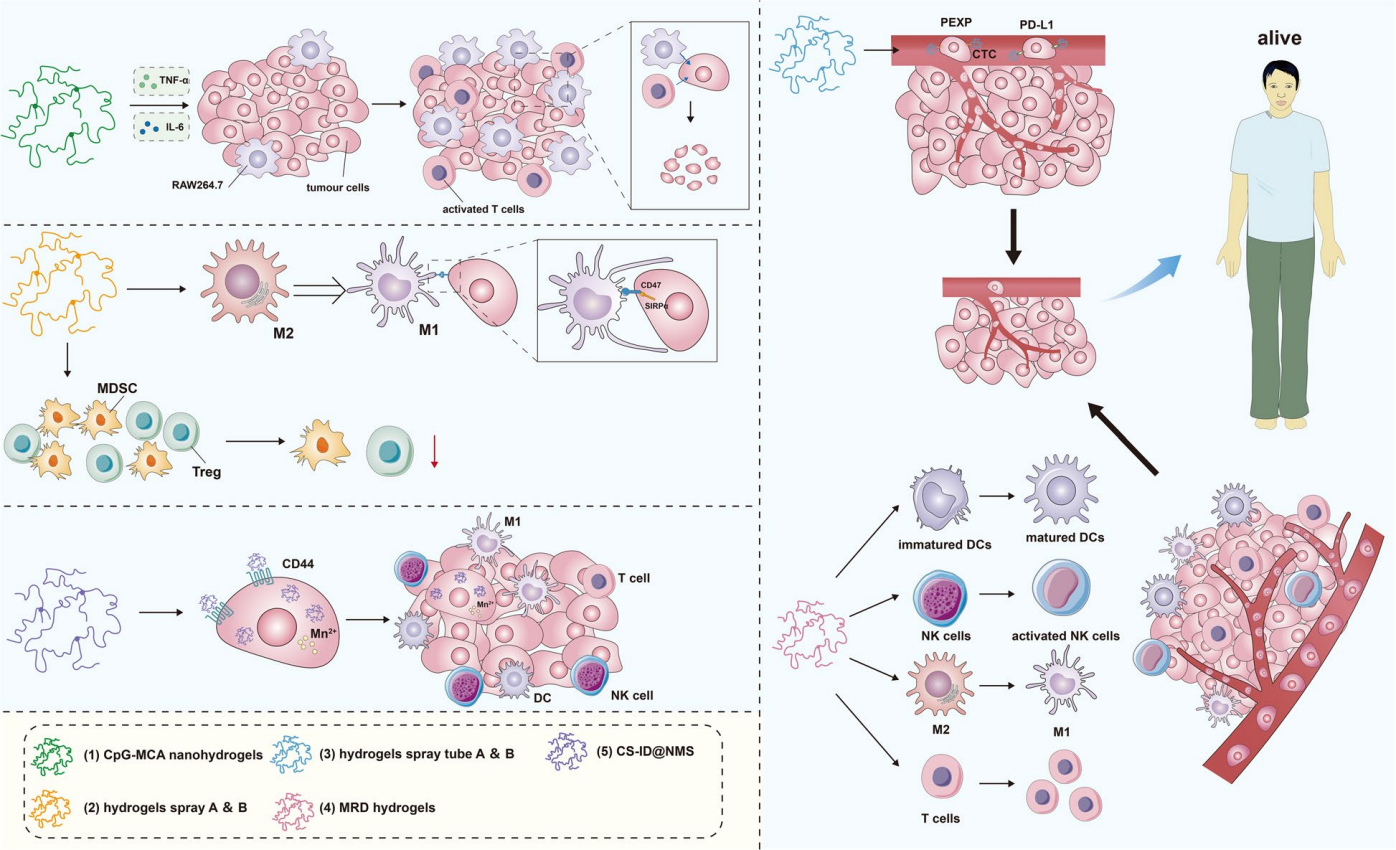
A summary of nano-hydrogel used in tumor immunotherapy to inhibit tumor progression. The 5 kinds of nano-hydrogel mentioned in this manuscript activate T cells and NK cells, inhibit MDSC and Treg cells, promote the transformation from M2 macrophage to M1 macrophage and restore tumor immunity to inhibit tumor progression

Oral hydrogel is a relatively new strategy in tumor immunotherapy [169, 170]. Xiao et al. constructed the SDT-based oral hydrogel nanomotors (NMs) CS-ID@NMs [171] which are not only non-invasive, but also exhibit great tissue penetration ability. Furthermore, CS-ID@NMs also induce ICD and activate the immune responses in tumor cell clearance. CS-ID@NMs also hugged the targeted ability of CD44, which is highly expressed in colon cancer cell membranes, to inhibit the ability of tumor invasion and migration [172–175]. NMs are released from the CS-ID@NM/ hydrogel upon oral administration, allowing them to penetrate the mucus layer and move into tumor tissues. Subsequently, these NMs are internalized into colon cancer cells via CD44-mediated endocytosis. IDs then rapidly release from these NMs and preferentially accumulate in cancer cells, immediately followed by the Mn^2+^-mediated killing of tumor cells and the generation of tumor debris to activate subsequent immune responses. A summary of the above-mentioned nano-hydrogel used in tumor immunotherapy to inhibit tumor progress was shown in Fig. 7.

### Liposomes

**Fig. 8:**
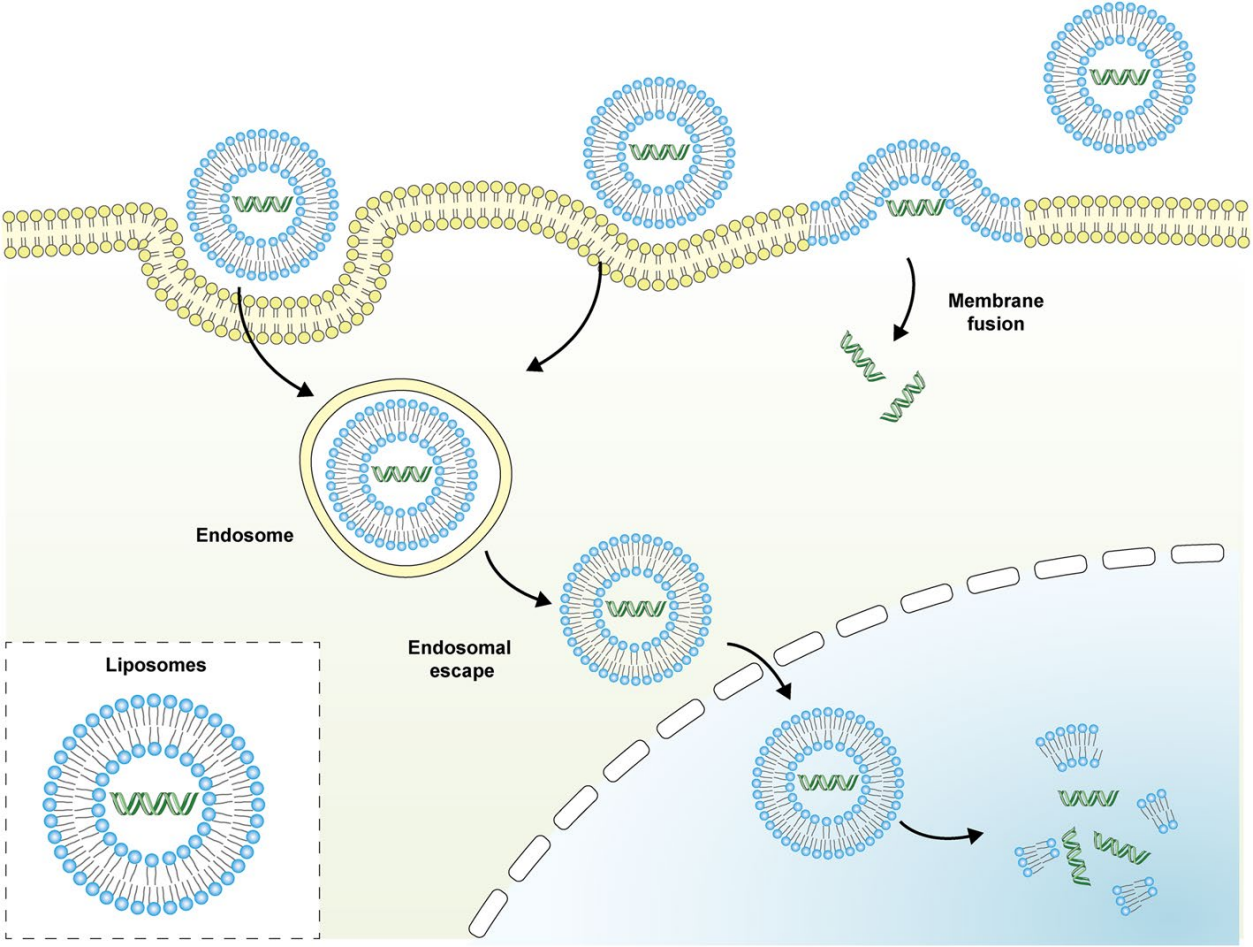
The basic action modes of liposomes. The contents of liposomes can enter tumor cells by membrane fusion and endocytosis. For membrane fusion, the contents can release into cytoplasm directly. For endocytosis, liposomes can induce the process of endosomal escape, then the contents release into cytoplasm and nucleus

Liposomes are spherical vesicles with double membrane layers. The double membrane layers are formed spontaneously by phospholipids dispersed in aqueous media. The amphiphilic nature of these liposomes makes them excellent drug carriers [176–180]. Liposomes have excellent biocompatibility, non-immunogenicity and biodegradability, all of which contributed to their strengths in targeted delivery and sustained release of loaded drug. Besides that, liposomes could also improve the therapeutic effect of the loaded agents by improving drug stability, enhancing drug solubility and reducing drug toxicity [181–183]. The basic action modes of liposomes were shown in Fig. 8.

**Fig. 9:**
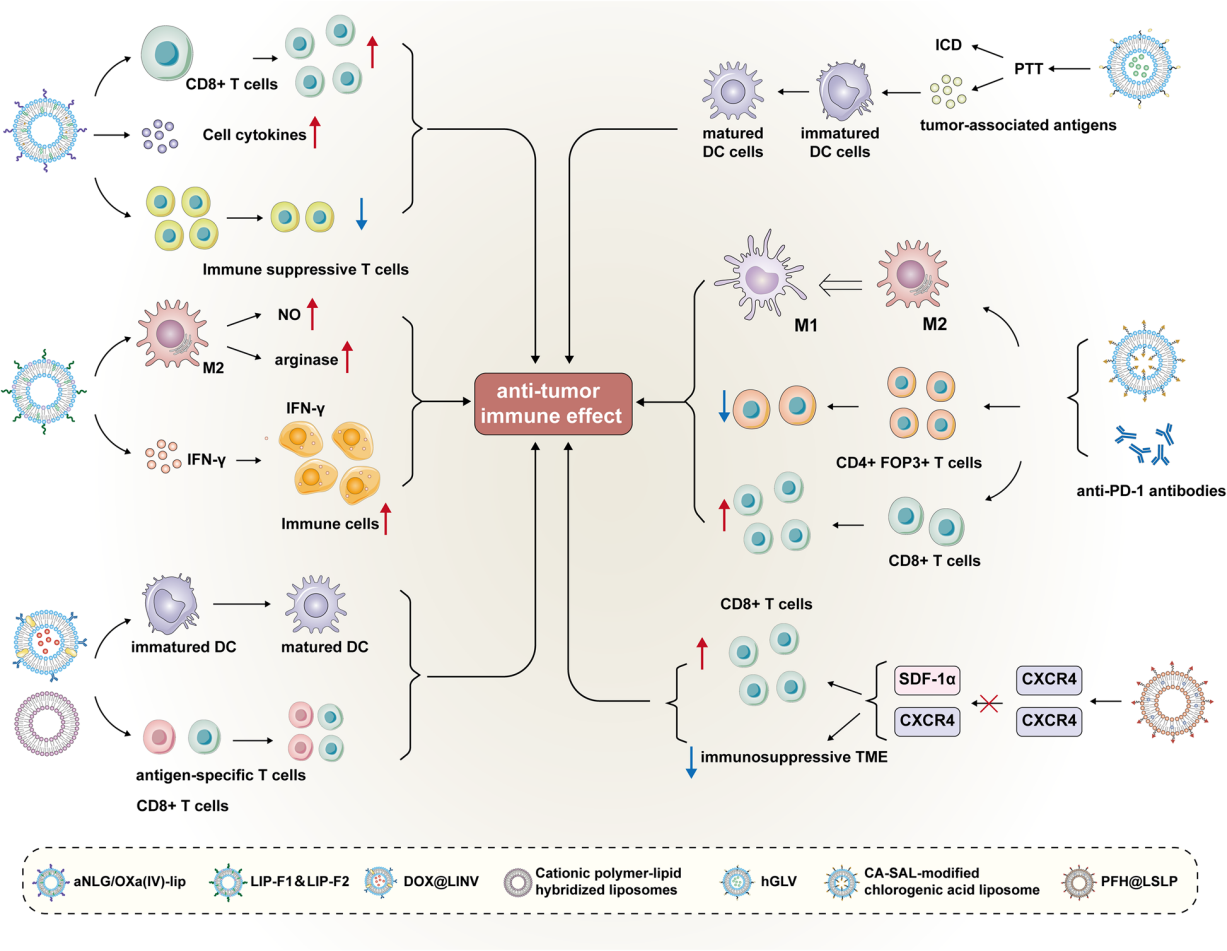
A summary of liposomes used in tumor immunotherapy to inhibit tumor progression. The 7 kinds of liposomes mentioned in this manuscript increase the infiltration of CD8^+^ T cells, decrease the level of immune suppressive T cells, promote the maturation of DCs, and induce the macrophage transformation from M2 type to M1 type and restore tumor immunity to inhibit tumor progression

Fengyun Shen et al. designed and developed a liposome aNLG/ Oxa(IV)-Lip via self-assembling oxaliplatin prodrug (Oxa(IV)). Oxa(IV) was conjugated with phospholipids and alkylated with NLG919 (aNLG) and other related commercial lipids. This novel liposome showed anti-tumor effect by increasing the infiltration level of CD8^+^ T cells, inducing the secretion of cytotoxic cytokines and decreasing the immunosuppressive T cells ratio [184]. Maryam Kateh Shamshiri et al. constructed a non-polyethylene glycolized (HSPC/DSPG/Chol, LIP-F1) liposome and a polyethylene glycolized (HSPC/DSPG/Chol/mPEG2000-DSPE, LIP-F2) liposome, which contained IFN-γ. Treating M2 macrophage with these novel liposomes can effectively increase the level of nitric oxide (NO) and reduce the level of arginase. These liposomes can also induce a strong anti-tumor immune response by increasing the level of IFN-γ transported into immune cells [185]. Mei Hu et al. designed and developed a novel liposome DOX@LINV. DOX@LINV was constructed by fusing artificial liposomes with nanovesicles derived from related tumor. After that, this fused liposome was loaded with DOX. This novel fused liposome DOX@LINV can stimulate the maturation of DCs and induced anti-tumor immune response of antigen-specific T cell, as a result, to perform anti-tumor effect in lung cancer, breast cancer and melanoma [186]. Qi Su and et al. constructed the cationic polymer-lipid hybridized nanovesicles which could significantly stimulate the maturation of DCs and increase the vaccine uptake ability of DCs. Furthermore, this novel liposome also increases the infiltration level of CD8^+^ T cells and drained the tumor lymph nodes, which predicted it can be a benign therapeutic method for tumor immunotherapy [187]. Jinbo Li et al. developed a liposome which can load paclitaxel, anthracyclines mitoxantrone or DOX. This novel liposome can induce ICD and tumor cell-killing to inhibit the development of tumor [188]. Lili Cheng et al. designed and developed a therapeutic nanovesicle called hGLV. hGLV was developed via fusing engineered exosomes with agent-loaded liposomes. CD47 was overexpressed in hGLV and can be used to change the mode of macrophage-mediated tumor cell phagocytosis by inhibiting the CD47 related signaling. Additionally, after intravenous injection, photothermal agents loaded in hGLV can promote superior PTT and complete elimination of tumor under the action of laser irradiation, which can also lead to ICD and promote the production of a lot of tumor-associated antigens to stimulate the maturation of DCs [189]. Ji Eun Won et al. developed a sialic acid (CA-SAL)-modified chlorogenic acid liposome. Then, this liposome was combined with anti-PD1 antibodies to be used in tumor immunotherapy of patients with melanoma. The combination can effectively decrease the ratio of M2 macrophage and CD4tFop3t T cells, and significantly increase the ratio of CD8^+^ T cells and M1 macrophage accompanied by activation of T cells. The combination also demonstrated synergistic anti-tumor effects to serve as a novel therapeutic method for patients with melanoma. They also developed chitosan hydrogels (CH-HG) containing gold cluster-labeled DOX liposomes (CH-HG-GL DOX). CH-HG-GL DOX could effectively inhibit the growth and survival of tumor and decrease the cell toxicity of DOX in vivo. When CH-HG-GL DOX was combined with poly (D, L-propylene-co-ethanolate) nanoparticle-based vaccines, they can have synergistic anti-tumor therapeutic effects [190]. Yuehua Wang et al. developed a liposome with an oxygen-saturated perfluorohexane (PFH) core and also modified the CXCR4 antagonist LFC131 peptide on the surface of the liposome. Besides, the liposome was used to deliver sorafenib and the CSF1/CSF1R inhibitor PLX3397 (PFH@LSLP) in HCC, which showed excellent therapeutic effects. PFH@LSLP could recognize hypoxia-related CXCR4 overexpression, block the signal SDF-1α/CXCR4 axis to activate anti-tumor immune response of CD8^+^ T cell and reverse immunosuppressive TME, finally prevent the development of HCC [191]. A summary of the above-mentioned liposomes used in tumor immunotherapy to inhibit tumor progress was shown in Fig. 9.

### Lipid nanoparticles

Lipid nanoparticles (LNPs), an emerging nanomaterial with excellent drug delivery ability, has been extensively explored in the area of tumor therapy [192, 193]. The most widely used LNPs included solid lipid nanoparticle (SLN) and nanostructured lipid carrier (NLC). The advantages of LNPs included biocompatibility, controlled and sustained release of anti-tumor drugs, and lower systemic toxicity, especially hugged the advantage to delivery mRNA for tumor immunotherapy [194, 195]. For the components of LNPs, typical LNPs were composed of cationic lipids, ionizable lipids, or helper lipids. Polyethylene glycol (PEG) lipids or surfactants may be also added to improve colloidal stability of the LNPs [196, 197]. At present, the potential clinical application of LNPs mainly lies in effectively modulating mRNA delivery to improve the immunotherapeutic effects. Naked mRNA was unstable, and can be rapidly degraded by nucleases and self-hydrolysis. Encapsulation of mRNA by LNPs could protect naked mRNA from extracellular ribonucleases and contributed to the intracellular mRNA delivery. As a result, the therapeutic effect of these mRNA can be fully performed. Many studies have convinced this point [198–201].

First, the LNPs vector have the adjuvant effect. LNP itself can activate the immune response [202]. Particularly, LNPs can contain cationic lipids, like 1,2-dioleyl-3-trimethylammonium-propane chloride salt, which could activate Toll-like receptor 4 (TLR4) and induce the secretion of pro-inflammation cytokines like IL-2, IFN-γ and TNF-α. A high level of monocyte infiltration also happened after injection of LNPs containing GFP mRNA [203]. Additionally, one novel kind LNP, called PL1, has been applied to effectively deliver the mRNA of costimulatory receptor CD134 or OX40 to tumor-infiltrating T cells to reactivate the anti-tumor immune response. Besides, combined PL1-OX40 mRNA with anti-OX40 antibody presented the higher anti-tumor ability compared with anti-OX40 antibody alone in a number of tumor models [204]. Furthermore, Oberli MA et al. further developed a LNP formulation for the delivery of mRNA vaccines to induce the cytotoxic CD8^+^ T cell response [205]. In this study, they treated B16F10 melanoma tumor using the LNP contained mRNA which coded for gp100 and TRP2. gp100 and TRP2 were two kinds of tumor-associated antigens [206]. Then, they found that the LNP formulation can cause tumor shrinkage and prolong the survival time of the model mice. Further analysis convinced that the transfection of DC, macrophage and neutrophils was performed using this LNP formulation, then cytosolic antigen synthesis and degradation enabled the antigen presentation on MHC-I. As a result, a strong cytotoxic CD8^+^ T cell anti-tumor immune response was activated. In addition, Chen J et al. also found that LNPs-mediated lymph node (LN) targeting delivery of mRNA vaccines induced robust CD8^+^ T cell response [207]. In this research, they developed an LNP formulation named 113-O12B. The targeted delivery of OVA-encoding mRNA vaccine to LN using 113-O12B significantly increase the CD8 + T cell response, the ratio of M1/M2 like macrophages and the therapeutic effect of the OVA-encoding mRNA vaccine in B16F10 melanoma model. Moreover, 113-O12B which was encapsulated with TRP2 peptide (TRP2180–188)–encoding mRNA also presented excellent tumor inhibition effect and improved the complete response (CR) to these tumor models, especially when combined with anti-PD-1 therapy. Above results proved that mRNA vaccine delivery using LNPs could effectively contribute to tumor immunotherapy in vivo.

Besides the mRNA of tumor associated antigens can be delivered by LNPs, anti-tumor cytokines mRNA can also be delivered. Liu JQ et al. performed direct intra-tumoral delivery of IL-12 and IL-27 mRNA using LNPs to assist in tumor immunotherapy [208]. They synthesized ionizable lipid materials containing di-amino groups called DAL4-LNP. Intra-tumoral injection of DAL4-LNP loaded with IL-12 or IL-27 mRNA significantly inhibited tumor growth of B16F10 melanoma. Most importantly, the delivery of IL-12 or IL-27 mRNA using DAL4-LNP also significantly induced immune effector cells infiltration into tumor, including IFN-γ and TNF-α produced NK cells and cytotoxic CD8^+^ T cell. Furthermore, the delivery did not cause significantly toxicity. Kheirolomoom A et al. also performed T cell transfection by anti-CD3-conjugated LNPs in situ, which leaded to T cell activation, migration and phenotypic shift to perform anti-tumor immune response [209]. They developed anti-CD3-targeted lipid nanoparticles (aCD3-LNPs) to deliver reporter gene mRNA which was specifical to T cells. aCD3-LNPs can activate > 85% of splenic CD4^+^ and > 90% of splenic CD8a^+^ T cells. Furthermore, aCD3-LNPs treatment significantly increased the level of pro-inflammation factors, like IFN-γ, IL-2, IL-3, TNF-α, IL-4, IL-5, IL-9 and IL-13 [210, 211].

LNPs have been widely used as a delivery method, especially for mRNA vaccine delivery in tumor immunotherapy. And there was no reported serve adverse effect when using LNPs in vivo validation. Furthermore, some personalized mRNA vaccines encoding various kinds of cancer-associated antigens have been formulated in LNPs and entered clinical stages [200, 212]. We do have the confidence that LNPs can be widely applied in clinical tumor immunotherapy in the future.

## Tumor immunotherapy based on nanoemulsion, a type of fabrication method for organic nanoparticles

Nanoemulsions (NEs) belong to colloidal dispersion system and were stabilized by surfactants. The average particle size of NEs was 20—200 nm [213–216]. Unlike conventional emulsions, NEs have smaller particle size, and better interfacial properties, transparency and kinetic stability [217, 218]. The biological efficacy and physicochemical stability of bioactive ingredients can be significantly improved by encapsulation into NEs [219–221].

**Fig. 10:**
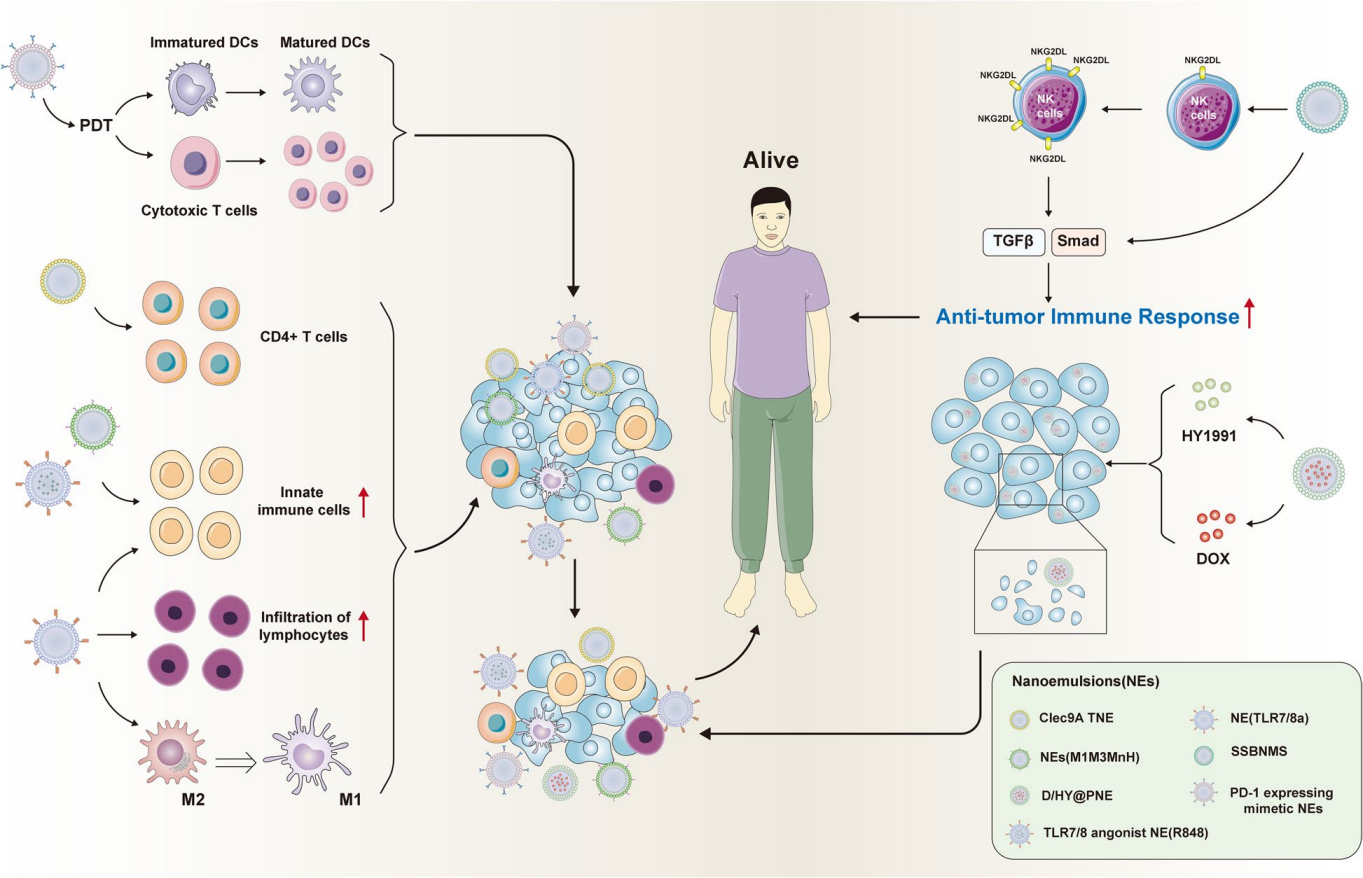
A summary of NEs used in tumor immunotherapy to inhibit tumor progression. The 7 kinds of NEs mentioned in this manuscript promote DCs maturation, increase CD4^+^ T cells infiltration, induce the macrophage transform from M2 type to M1 type and restore tumor immunity to inhibit tumor progression

Bijun Zeng et al. designed and developed a novel functionalized custom NEs which encapsulate tumor related antigens to target Clec9A (Clec9A TNE). Clec9A TNE encapsulated six neoepitopes and significantly inhibited the growth of B16-F10 melanoma with the help of CD4^+^ T cells [222]. Sun-Young Kim et al. developed a local in situ vaccination with tumor antigens adjuvanted with NE loaded with TLR7/8 agonists [NE (TLR7/8a)]. This vaccines adjuvant with NE can significantly induce the activation of innate immune cells, increase the infiltration level of lymphocytes, and promote polarization of M2 macrophages, which contributed to inhibiting tumor growth and improving survival outcomes in mice with tumor [223]. Xiumin Zhang et al. constructed a composite protein vaccine encapsulated by NEs (M1M3MnH) by magnetic ultrasound technology. NEs (M1M3MnH) can induce a stronger anti-tumor immune response than that by M1M3MnH, NE(-) or PBS, indicating the strong anti-tumor immune effect of this novel NEs carrier on encapsulated antigen [224]. Chang Liu et al. constructed a NEs system (SSB NMs) to co-deliver TGF-β inhibitors and selenocysteine (SeC) for enhancing immune response of T cells. SSB NMs enhanced the surface expression level of NKG2DL on NK92 cells, which subsequently boost anti-tumor immune response via the TGF-β/TGF-β RI/Smad signaling pathway. Furthermore, SSB NMs may release TGF-β inhibitors and SeC in a continuous manner, and act with NK92 cells to produce excellent anti-tumor immune response synergistically [225]. Le Jia et al. designed a NEs (PNE)-D/HY@PNE to deliver ICD inhibitor HY19991 (HY) and DOX into specific sites. D/HY@PNE can rapidly release HY and DOX-loaded nanogels which enhance penetration ability and ICD induction, ultimately improving antitumor effect in mice with 4T1 tumor [226]. Yifan Zhang et al. designed and developed a PD-1-expressing mimetic NEs. In this NEs, perfluorinated carbons could supply oxygen to against hypoxic tumors and also serve as a source of Photo Dynamic Therapy (PDT). Furthermore, this PD-1-expressing mimetic NEs can deliver PD-1 proteins and photosensitizers together and contribute to a synergistic effect of PDT and immunotherapy. Then PDT may inhibit growth and metastasis of tumor via stimulating DC maturation and increasing the infiltration level of cytotoxic T lymphocytes [227]. Jiae Koh et al. designed a vaccine which contained the TLR7/8 agonist NE (R848). This vaccine can induce the anti-tumor immune response via activation of T cells and inhibition of T cell depletion, and prevent recurrence and metastasis of tumor to improve the survival outcomes of mice with tumor [228]. Besides, Rodrigues MC et al. also developed aluminum phthalocyanine-mediated NEs (PDT-AlPc-NE). This PDT-AlPc-NE can effectively inhibit the tumor growth and lung metastasis in a murine model with 4T1 breast adenocarcinoma [229, 230]. A summary of the above-mentioned NEs used in tumor immunotherapy to inhibit tumor progress was shown in Fig. 10.

## Tumor immunotherapy based on inorganic nanomaterials

Inorganic nanomaterials possess controllable shape and size, well-defined chemical properties, and excellent optical, electrical and magnetic properties with huge advantages for tumor immunotherapy [231–233]. Inorganic nanomaterials mainly include nonmetallic and metallic nanomaterials.

### Non-metallic nanomaterials

The nano-vaccine OVA@SiO2 was constructed by preparing SiO2 solid nanospheres and covalently binding OVA to activated SiO2 surface. OVA@SiO2 could stimulate the maturation of bone marrow-derived DCs [234]. Hollow mesoporous silica nanoparticles (H-XL-MSNs) with extra-large mesopores were also constructed. The application of PEI solution on the surface of H-XL-MSNs could promote the maturation of DCs and significantly increased the ratio of cytotoxic T cells, ultimately inhibiting the growth and survival of tumors [235]. Yi-Ping Chen et al. used PEG, ammonium-based cationic molecules (TA), modified rhodamine B isothiocyanate (RITC) and fluorescent mesoporous silica nanoparticles (MSN) to construct cdG@RMSN-PEG-TA. The secretion of IL-6, IL-1β, and IFN-β were stimulated when RAW 264.7 cells were treated with cdG@RMSN-PEG-TA. Besides, the protein expression of phosphorylated STING (Ser365), CD11c^+^ dendritic cells infiltration, F4/80^+^ macrophages, and CD4^+^ T cells and CD8^+^ T cells were all enhanced as well, ultimately inhibiting malignant development of breast cancer [236]. Peiqi Zhao et al. loaded tumor acidic environment-responsive CCM camouflaged mesoporous silica nanoparticles (CMSN) with dacarbazine (DTIC) and bound it to aPD1. DTIC@CMSN can effectively inhibit growth and metastasis of melanoma by inducing anti-tumor immune response of T cells [237]. Linnan Yang et al. used polyethyleneimine-modified dendritic silica nanoparticles which was loaded with microRNA-125a to construct DMSN-PEI@125a. DMSN-PEI@125a could promote M1 polarization of tumor-associated macrophages and increase the infiltration level of NK cells and CD8^+^ T cells to inhibit growth and metastasis of breast cancer [238]. Xiupeng Wang et al. presented that HMS nanospheres promoted maturation of DCs. Additionally, HMS-based cancer vaccines showed synergistic effects with anti-PD-L1 antibodies on tumors to effectively increase the level of CD4^+^ and CD8^+^ T cell, ultimately inhibiting development of tumor [239].

### Metallic nanomaterials

Phospholipid coated amorphous porous manganese phosphate drug nanocarrier (PL/APMP-DOX) is a Mn-based hybrid nanoparticle that have been applied in tumor immunotherapy [240, 241]. Owing to modifications done on its surface phospholipids, the drug nanocarrier PL/APMP-DOX remains intact in the blood in vivo and can be broken down in 4T1 mouse breast cancer cells which express high level of phospholipid. APMP-DOX will then be further disintegrated, where Mn^2+^ and DOX are released into the intracellular microenvironment. Released DOX results in DNA leakage, which subsequently activates the GMP-AMP-cGAS-STRING pathway. Together with activity of the Mn^2+^-enhanced STRING pathway, PL/APMP-DOX promotes the production of type I-interferon, maturation of dendritic cells, and increased the population of cytotoxic T lymphocyte or natural killer cells for increased cytotoxicity effect in killing tumor cells. In addition, PL/APMP-DOX could also greatly enhance the secretion of pro-inflammatory cytokines and inhibit the progression of tumors. Besides, CaP can be used to coat the outer membrane vesicles (OMVs) of bacteria, inciting powerful immune-stimulant ability. The pH-sensitive CaP shells facilitate the polarization of macrophages from M2 to M1 to assist in anti-tumor immunity [242]. Moon et al. also constructed a self-assembled nanoparticle CMP based on the Mn^2+^ and CDN STING agonist. CMP_CDA_ was also prepared which could significantly enhance the activity of STING agonist IFN-I [243]. In vitro experiments showed that CMP_CDA_ can be easily taken by BMDCs and it promotes the secretion of IFN-β by BMDCs. Resultsfrom in vivo experiment suggested that CMP_CDA_ significantly inhibits tumor growth by elevating the levels of IFN-β, TNF-a, CXCL10 and CCL5, and enhancing CD8^+^ T cell responses in tumor-bearing mouse.

Aluminum phosphate nanoparticles was used to coat mouse melanoma B16-F10 tumor cell membrane and CpG to construct the APMC nanoparticles [244]. Aluminum phosphate is an immune adjuvant with high safety profile, and it has been used in clinical adjuvant treatment. The binding of aluminum phosphate and CpG activates cell immunity reaction [245, 246]. Besides, B16-F10 tumor cell membrane in APMC provides many kinds of tumor-associated antigens and ascertained the mobility and stability of APMC. Results from in vivo experiments also found that APMC increases the activity of CD4^+^ T cells, CD8^+^ T cells and cytotoxic T cells, besides increasing the release of inflammation factors in the lymph node. As a result, tumor growth was inhibited and survival was prolonged in mouse model receiving APMC.

Zn^2+^ was also used to wrap nanoparticles. Luan et al. constructed a Zn^2+^ based nanoparticles which could disrupt myeloid-derived suppressor cells (MDSCs)-induced immunosuppression [247]. Zn^2+^ based pH-responsive ZIF-8 (zeolite imidazolium framework-8) was used as a carrier for the nanoparticle. By loading the drug mitoxantrone and DNA demethylating agent Hydralazine, (M + H) @ZIF was obtained. Eventually, (M + H) @ZIF with a hydrated particle size can be obtained by modifying (M + H) @ZIF/HA with a kind of negatively charged HA. MDSCs-induced immunosuppression was achieved by affecting the normal tumor-killing function of T cell via MDSCs specific metabolite MGO [248, 249]. After being treated by (M + H) @ZIF/HA, the concentration of MGO showed a significant decreased in MDSCs, thus predicting the alleviation of MDSCs inhibition on normal T cell functions. Researchers also noticed the ability of (M + H) @ZIF/HA to promote DCs maturation and increase the infiltration level of cytotoxic T cells and helper T cells at tumor site, transforming the “cold tumor” into “hot tumor” [250, 251]. As a result, both growth and metastasis rates of the tumor were attenuated. Tan et al. also wrapped ligand-driven self-assembly of DNA and metal ions into MXFs nanomaterials Hf-CpG MXF [252]. Results from in vitro assays showed that Hf-CpG MXF effectively enhanced radiotherapy-induced tumor cell DNA damage, promoted cell differentiation of mature DCs, and induced CD8^+^ and CD4^+^ T cell infiltration in distal tumor tissues. Besides, the combination of radiotherapy and Hf-CpG MXF nanomaterials significantly inhibited tumor growth and prolonged the survival of CT26 tumor-baring mice.

## New strategies and challenges

These mentioned novel nanomaterials in this review provided new means of drug delivery with unique advantages such as controlled release and improved biosafety due to their optical, magnetic and electrical properties [253–257]. Besides, high biocompatibility and lower systemic toxicity also further promote the widely application of nanomaterials in the area of tumor therapy [258, 259]. Furthermore, more and more studies have convinced that novel nanomaterials such as LNPs, can not only inhibit the development of tumor by effectively delivering mRNA vaccines, but also can serve as an adjuvant to activate the anti-tumor immune response directly [202, 260]. The rapid update of nanomaterials-based methods has paved the way for the development of highly effective approaches for tumor treatment in clinical practice.

Meanwhile, in the past decades, although great progress has been made in tumor diagnosis, chemotherapy, and targeted therapy [261–264], tumor drug resistance and severe toxic side effects of treatments are still the major challenges faced by clinicians in clinical practice [265–267]. However, the rapid development of tumor immunotherapy has changed the landscape of tumor treatment in various solid tumors and hematologic malignancies because unlike the methods presented above, tumor immunotherapy kills tumor cells by rebuilding anti-tumor immune response, persisting the response or raising the immune response threshold directly. Tumor immunotherapy significantly prolonged survival time among patients and improved the quality of life of patients with tumors, especially those with advanced stage of tumor and those who does not respond well to routine treatment methods [53, 268, 269]. Nonetheless, the efficacy of tumor immunotherapy varies, and immune response reactivation only occurs in a relatively small subset of patients with tumors [51]. Moreover, the lack of biomarkers to accurately gauge therapy efficacy remains the biggest challenge to achieve personalized immunotherapy in tumor precision medicine, since the patient’s response to single-agent immunotherapy or even multi-agents combined immunotherapy cannot be assessed [59, 270–272].

Although several antibodies which target CTLA-4 (such as ipilimumab) [64] and PD-1/PD-L1 (such as atezolizumab [273] and pembrolizumab [274]) have been approved by the US FDA, and the emerging strategies like bispecific T cell engagers (BiTEs), trispecific T cell engagers (TriTEs) based nanobodies have been thought to be a huge gain for therapeutic efficacy [275, 276], many treatment issues were found as well [277–280]. For example, some common adverse effects include pruritus, rash, nausea, diarrhea and thyroid disorders. Besides, treatment with these antibodies also presented significant personalized differences, and the diseases that can be treated with CTLA-4 and PD-1/PD-L1 antibodies are limited to only several types of tumors, not every kind of tumors [281–283]. Clinical trials have also shown limited effectiveness of these antibodies in targeting other kinds of tumors [56, 284]. Both immune checkpoint inhibitors (monoclonal antibody) and adoptive cell transfer (CAR-T, CAR-NK and TCR-T therapies) required very specific and effective targets. However, even convinced tumor associated antigens which were necessary for restarting anti-tumor immune responses can also be blocked by polysaccharides, making them difficult to be processed for presentation and thus the tumor-killing effect cannot be achieved completely [9, 60, 285]. Meanwhile, the interactions between tumor cells and TME built a hypoxic, high-pressure and acidic environment which also affect the actions of immunotherapeutic drugs [13, 286–288]. In vitro and in vivo experiments have convinced that the application of novel nanomaterials in tumor immunotherapy could effectively solve the above drawbacks.

These novel nanomaterials could enhance treatment effects of tumor immunotherapy by targeting cancer cells, TME and even the immune system. Novel nanomaterials can also prolong the in vivo circulation time of drugs, improve the immunosuppressive tumor microenvironment and further activating tumor-killing T cells; meanwhile, it can effectively reduce toxicity, enhance permeability and retention effects of drugs, and minimize the shielding effect of protein corona [289–293]. Although a lot of effort has been put in to improve the efficacy of tumor immunotherapy, and progress has been made in the development of nanodrug delivery systems (NDDSs), no NDDSs have made it up to the clinical practice stage just quite yet [294–296]. This condition may result from various reasons. Firstly, the vast differences between tumors in mouse and human contribute to the discrepancy between the preclinical therapeutic effects of NDDSs and the outcomes of actual clinical trials. Therefore, the results of NDDSs clinical translations were not satisfactory [297, 298]. So, the development of humanized animal models which include human-derived immune systems and tumors would be an effective method to improve the efficiency of clinical translation for NDDSs research [299, 300]. Secondly, the enhanced permeability and retention (EPR) effect is still an important basis for achieving enrichment of NDDSs in tumors [301–304], but the uncertainty in terms of EPR effect in tumor tissues of different patients leads to uncertainty in the actual enrichment ability of NDDSs. This issue may be resolved by detecting the level of EPR effect in patients. Lastly, the mechanism of how NDDSs achieve active targeting remains unclear. Nevertheless, it is still strongly believed that by coupling the advances in cancer biology and immunology research with the development of novel NDDSs to improve drug-targeted delivery, the effectiveness of nanomaterial encapsulated drugs can be greatly enhanced while also achieving safer and effective tumor immunotherapy at the same time.

As for the new challenges, we must admit that there is still a long way to apply these useful findings into the real clinical practice. Although there are many pre-clinical studies that gained meaningful results [305–310], the available clinical trials with significant positive results were rare. The available clinical trials of nanomaterials-based tumor immunotherapy were also summarized in Table 3. The main limitation of current immunotherapy may be that most of the immunotherapy drugs fail to reactivate T cell and expand it in vitro or in vivo, and the occurrence of T cell exhaustion and adaptive immune resistance owing to various complex factors under the interaction between the tumor and TME. To overcome these issues, the most effective methods should be able to induce the formation of highly specific T cells and to prevent T cell exhaustion, which can be effectively achieved by combination therapy and utilization of novel nanomaterials for the co-delivery of immune stimulatory signals (agonists) and immune suppression inhibitors (antibodies). Some researchers have attempted to make some efforts. one study constructed a kind of iron nanomaterial modified with anti-CD137 and anti-PD-L1. Anti-CD137 provides co-stimulation signaling to enhance T cell proliferation and the specificity to target responding tumor cells, and anti-PD-L1 could block the interaction between PD-L1 and PD-1 to prevent PD-1 mediated T-cell exhaustion in melanoma [308]. Additionally, lipid nanoparticles-based delivery of mRNA vaccines could mimic the normal physiological process which T cell undergo and induce the anti-tumor immune response of the antigen-specific priming and boosting. One study designed a lipid nanomaterials-based mRNA vaccines (CARVac) to solve the issue of low persistence of CAR T cells in vivo. They featured responding CAR T cells targeting claudin 6 combined with this CARVac, a liposome encapsulating mRNA encoding claudin 6. Then, they found this CARVac leaded to the overexpression of claudin 6 on the surface of antigen-presenting cells and the release of co-stimulatory signals for effective CAR T cells priming and boosting. Ultimately, these T cells presented high specificity to directly targeting tumor cells [311]. From these reported results, we highly believe that combinational therapy and utilizing novel nanomaterials for the co-delivery of immunotherapy drugs have high potential to solve the current limitations and contribute to the rapid development of tumor immunotherapy in the real clinical practice. It is also our main research direction about the application novel nanomaterials in tumor immunotherapy in the future.

**Table 3:** Available clinical trials of nanomaterials-based tumor immunotherapy

Nanomaterials	Cargo molecules	Indications	Stage	Results	Reference
Cyclodextrin polymer-based nanoparticle	Small interfering RNA to reduce the expression of the M2 subunit of ribonucleotide reductase	Melanoma; Gastrointestinal cancer; Prostate cancer	Phase Ia/Ib	The pharmacokinetics of CALAA-01 in humans show fast elimination and reveal that the maximum concentration obtained in the blood after i.v. administration correlates with body weight across all species	[312]
Poly-ICLC, a double-stranded RNA complex that consists of poly(I:C) (polyinosinic: polycytidylic acid) stabilized by poly-L-lysine	cholesteryl pullulan and NY-ESO-1 antigen protein	advanced or recurrent esophageal cancer	phase I	Comparing CHP-NY-ESO-1 alone to the poly-ICLC combination, all patients exhibited antibody responses, but the titers were higher in the combination group	[313]
Poly-ICLC, a double-stranded RNA complex that consists of poly(I:C) (polyinosinic: polycytidylic acid) stabilized by poly-L-lysine	NY-ESO-1 antigen protein and montanide	high-risk resected melanoma	phase I/II	The combination with poly-ICLC is safe, well tolerated, and capable of inducing integrated antibody and CD4 + T-cell anti-tumor immune responses	[314]
Cholesteryl pullulan (CHP)-antigen protein nanoparticle	NY-ESO-1 antigen protein	esophageal cancer	phase I (NCT01003808)	The safety and immunogenicity of CHP-NY-ESO-1 vaccine were confirmed	[315]
Nanogels of cholesteryl pullulan	HER2 protein 1–146	Solid cancer expressed HER2	phase I	CHP-HER2 vaccine was well tolerated; HER2-specific CD8( +) and/or CD4( +) T-cell immune responses were detected	[316]
Nanoparticle albumin	Nanoparticle albumin-bound (nab)-paclitaxel & atezolizumab (anti-PD-L1 antibody)	Unresectable locally advanced or metastatic triple-negative	phase 3 trial (NCT02425891)	Nanoparticle albumin-bound (nab)-paclitaxel may enhance the anticancer activity of atezolizumab; the median progression-free survival was 7.5 months with atezolizumab plus nab-paclitaxel, as compared with 50 months with placebo plus nab-paclitaxel in patients with PD-L1 positive expression; the median overall survival was 25.0 months with atezolizumab plus nab-paclitaxel and 15.5 months with placebo plus nab-paclitaxel in patients with PD-L1 positive expression	[317]
Poly (beta-amino ester)-based nanomaterial	Plasmids encoding a 194-1BBz CAR and a piggyBac transposase	To be determined	Phase 1 projected 2020–2021	DNA-carrying nanoparticles can efficiently introduce leukaemia-targeting CAR genes into T-cell nuclei, thereby bringing about long-term disease remission	[318]
IL-15 super-agonist complex nanogel	IL-15 superagonist complex	Solid cancer and lymphomas	Phase 1	Nanogels delivery selectively expanded T cells 16-fold in tumors and allowed at least eight-fold higher doses of cytokine to be administered without toxicity in tumor microenvironment	[319]

In summary, this review mainly discussed the progress of developing novel nanomaterial-based tumor immunotherapy in the past few years. Meanwhile, we believe this study will provide an inspiring perspective for exploring new tumor immunotherapy strategies that will achieve a breakthrough in clinical practice.

## Data Availability

The data that support the findings of this study are available from the corresponding author upon reasonable request.
